# Supplementary material for: A Comparative Endocrine Trans-Differentiation Approach to Pancreatic Ductal Adenocarcinoma Cells with Different EMT Phenotypes Identifies Quasi-Mesenchymal Tumor Cells as Those with Highest Plasticity
Source: Cancers (Basel). 2021 Sep 17;13(18):4663. doi: 10.3390/cancers13184663 (PMC8466512; doi:10.3390/cancers13184663)
Supplement: Supplementary file 1 [file cancers-13-04663-s001.zip › cancers-1303182-supplementary.pdf]

Supplementary Material: A Comparative Endocrine Trans-Differentiation Approach to Pancreatic Ductal Adenocarcinoma Cells with Different EMT Phenotypes Identifies Quasi-Mesenchymal Tumor Cells as those with Highest Plasticity

Paula M. Schmidtlein, Clara Volz, Rüdiger Braun, Isabel Thürling, Olha Lapshyna, Ulrich F. Wellner, Björn Konukiewitz, Hendrik Lehnert, Jens-Uwe Marquardt and Hendrik Ungefroren

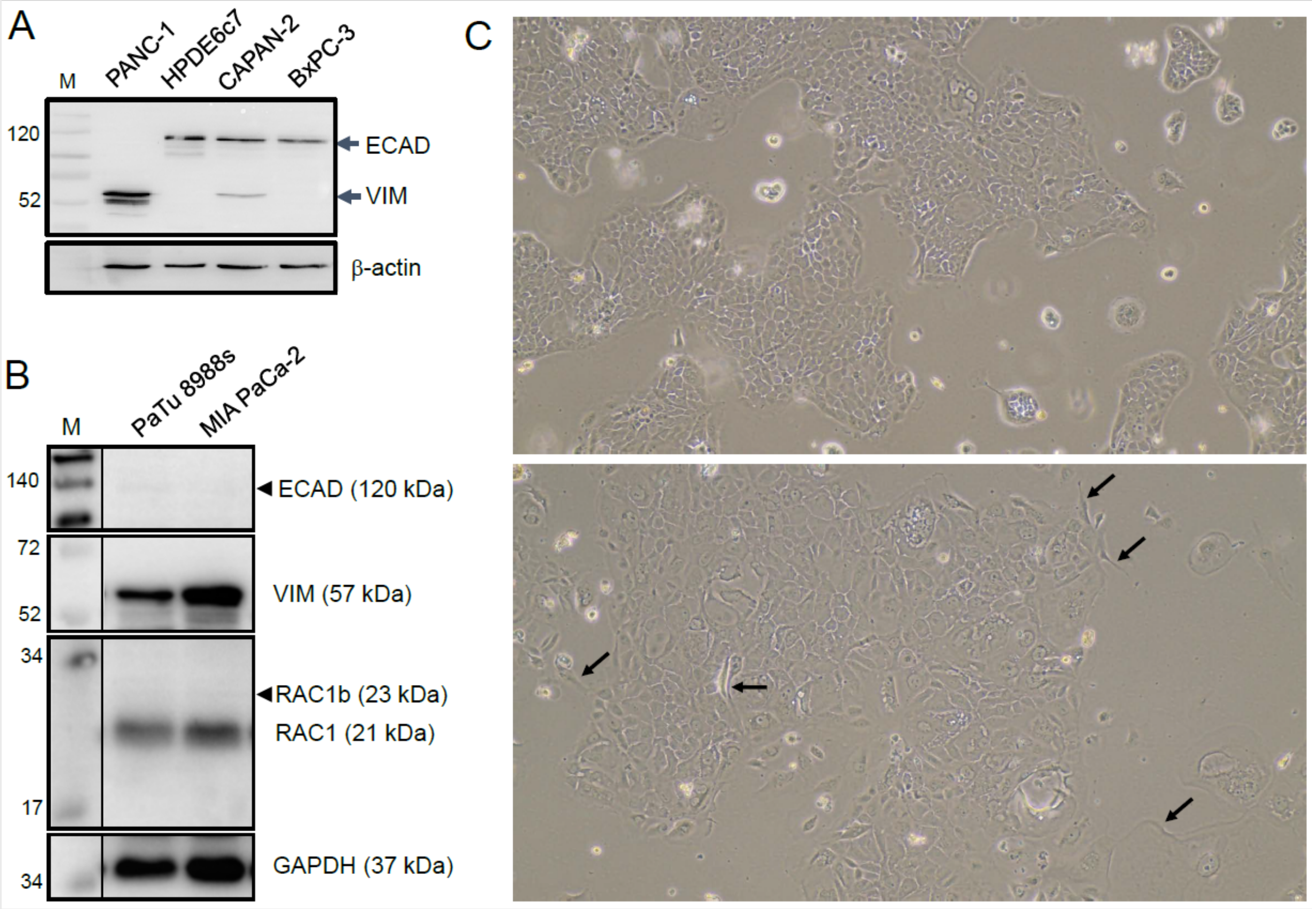

Figure S1. Immunoblot analysis of EMT markers in PaTu 8988s and MIA PaCa-2 cells, and growth pattern of LüPanc1 cells. (**A**) Short exposure of the same blot shown in Figure 1A. (**B**) Immunoblot analysis of EMT markers in PaTu 8988s and MIA PaCa-2 cells. Crude cellular lysates from the two PDAC cell lines were immunoblotted for ECAD, VIM, RAC1, the RAC1 splice isoform, RAC1b, and GAPDH as a loading control. The left lane shows relevant bands of the molecular weight marker (M) with the numbers next to them indicating their sizes in kDa. (**C**) Microscopic images of LüPanc1 cells to demonstrate their growth pattern. The arrows point to cells with elongated/spindle-shaped morphology. Whether some of these cells are senescent remains to be studied. Magnification, 100x.

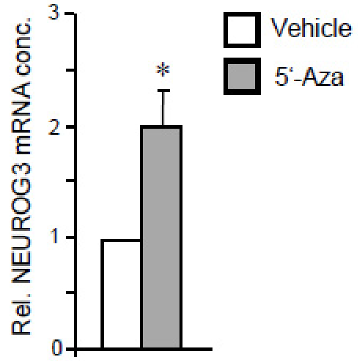

Figure S2. Epigenetic drug-induced trans-differentiation of primary PDAC-derived tumor cells to endocrine precursors. LüPanc1 cells were treated with 5’-Aza (1 μM), or vehicle (DMSO), in normal growth medium for 3 d followed by another 3 d in medium without 5’-Aza. Activation of the *NEUROG3* gene was assessed by qPCR analysis. Data are the mean ± SD of three independent assays (n = 3) with control cells set arbitrarily at 1.0. The asterisk (*) denotes a significant difference relative to the vehicle control ($p < 0.05$, Wilcoxon test).

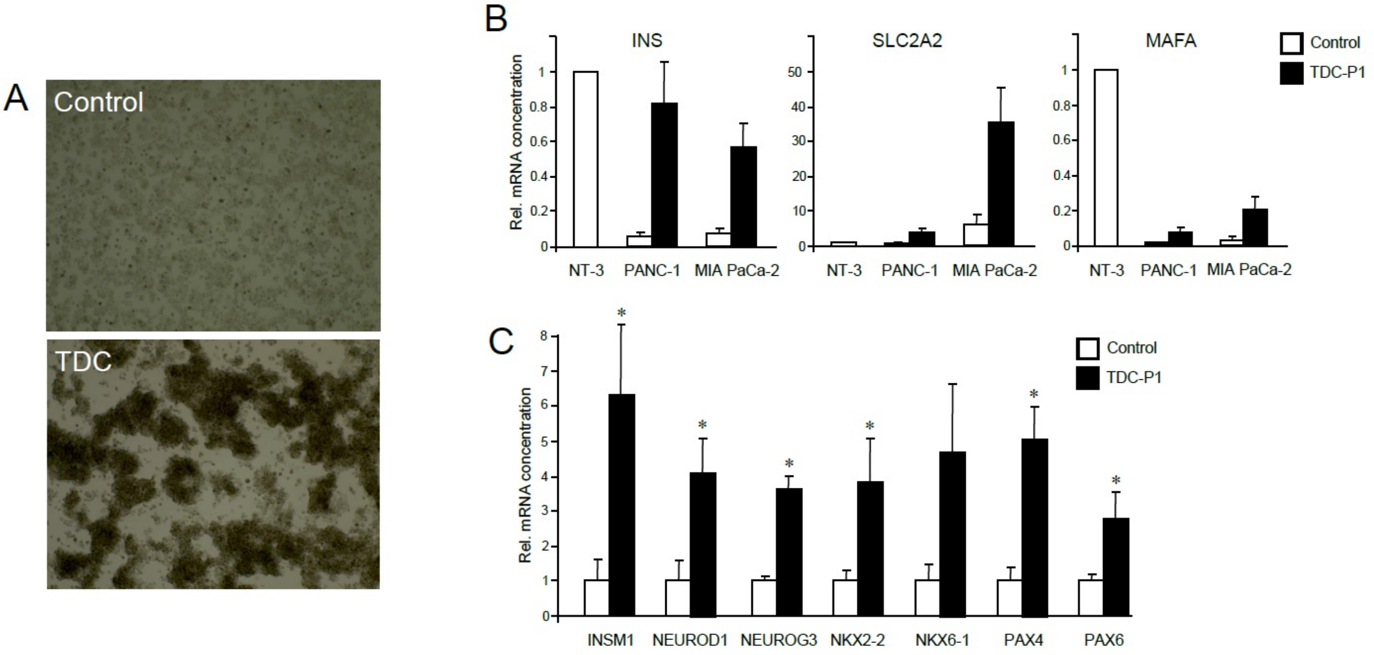

Figure S3. Trans-differentiation of MIA PaCa-2 and COLO 357 cells to pancreatic β cell-like cells. (**A**) Growth pattern of MIA PaCa-2 cells under control conditions and after TDC with FGF-b and transferrin (TDC-P1) for 5 d. Magnification x200. (**B**) Comparison of INS, SLC2A2 and MAFA mRNA levels, as measured by qPCR, in the PANC-1 and MIA PaCa-2 cell lines with those in the human insulinoma cell line NT-3. (**C**) QPCR-based detection in COLO 357 cells of the indicated markers of pancreatic endocrine development after a 7-d TDC-P1, or control medium. The data are representative of three assays (mean ± SD of triplicate wells). The asterisk indicates significance relative to controls set arbitrarily at 1.0. The asterisks (*) denote significant differences relative to the respective controls ($p < 0.05$, unpaired two-tailed Student‘s t test).

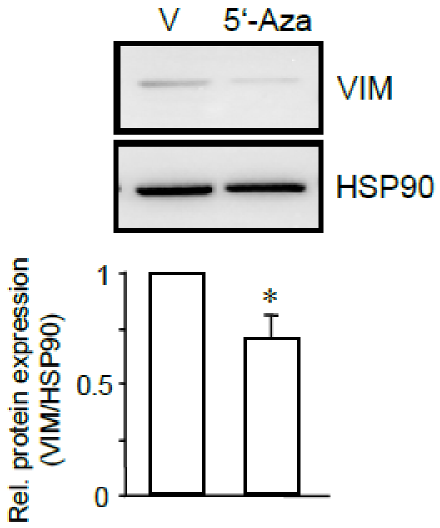

Figure S4. Immunoblot analysis of VIM in PANC-1 cells after treatment with 5′-Aza. PANC-1 cells were treated with 1 μM 5′-Aza, or vehicle (V), as outlined in the Material and Methods section and protein lysates subjected to immunoblotting of VIM. Detection of HSP90 served to verify equal gel loading. The graph underneath the blots depicts quantification of band intensities (mean ± SD, n = 3). A significant difference between groups is denoted by an asterisk (*).

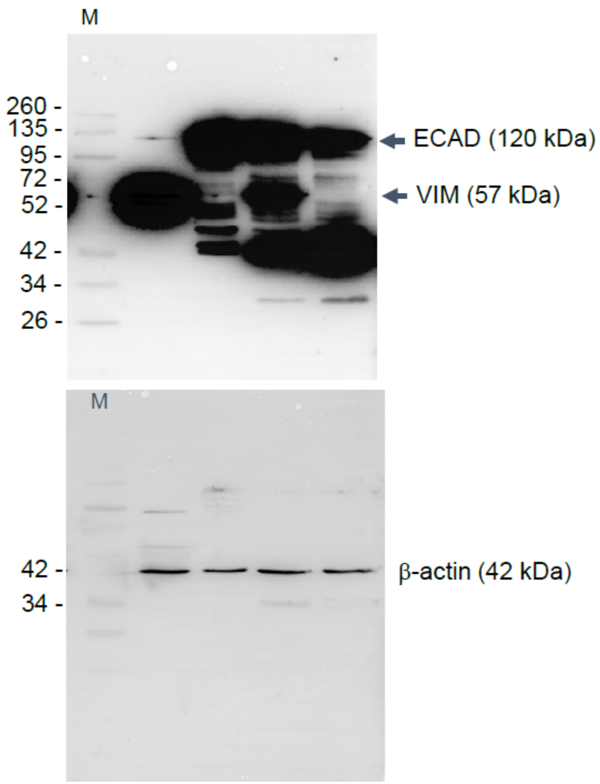

Figure S5. Uncropped blots of Figure 1A.

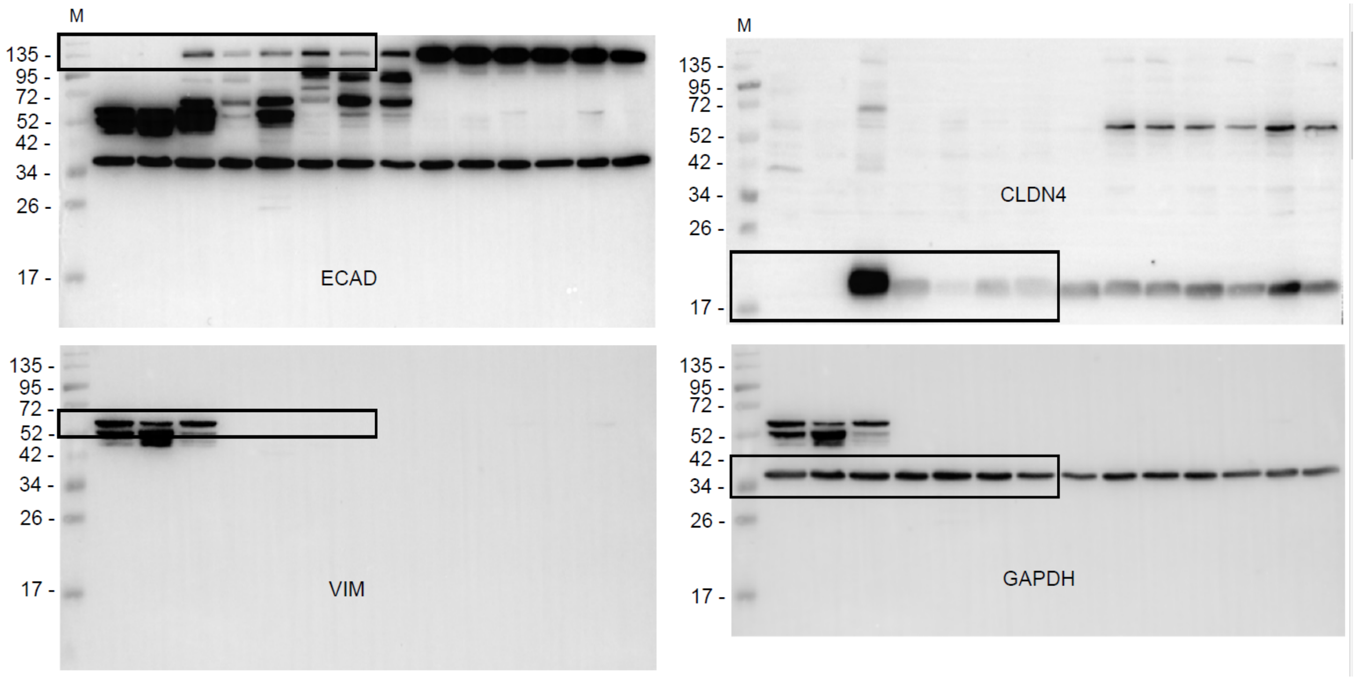

Figure S6. Uncropped blots of Figure 1B.

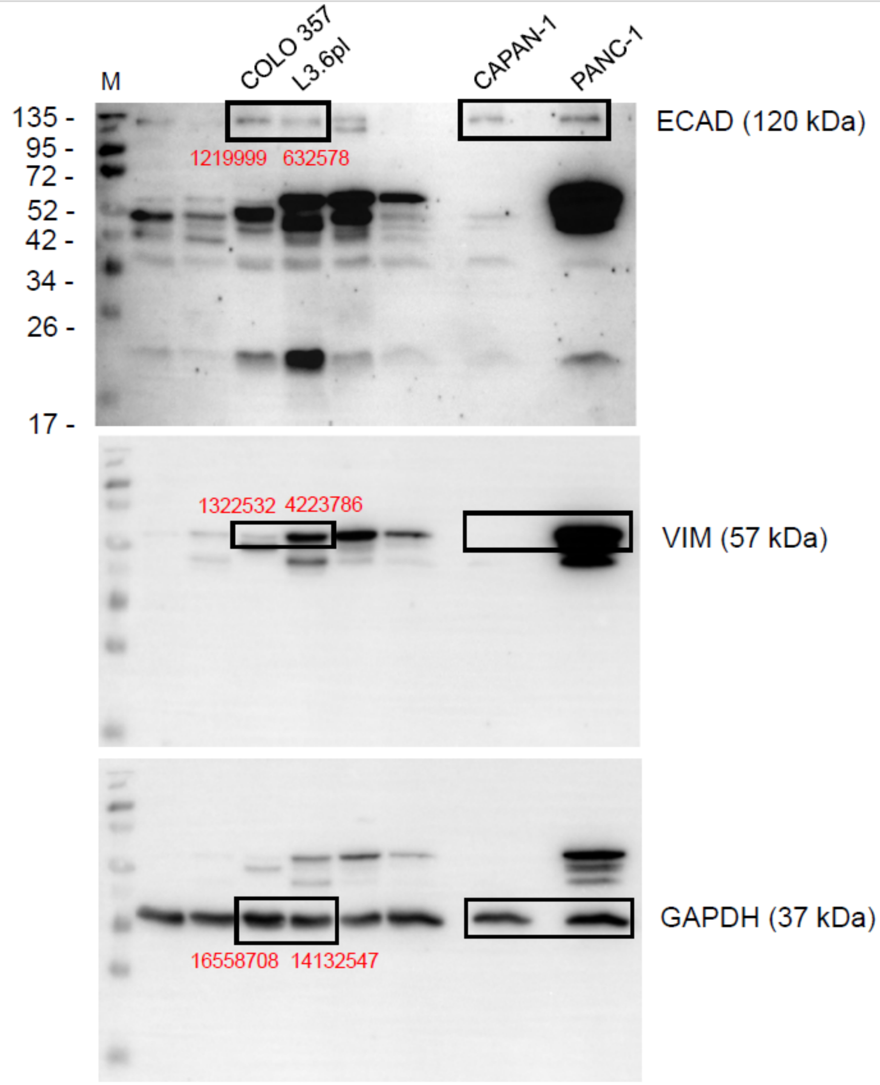

Figure S7. Uncropped blots of Figure 2A. Densitometric reading are given in red color below or above the respective bands.

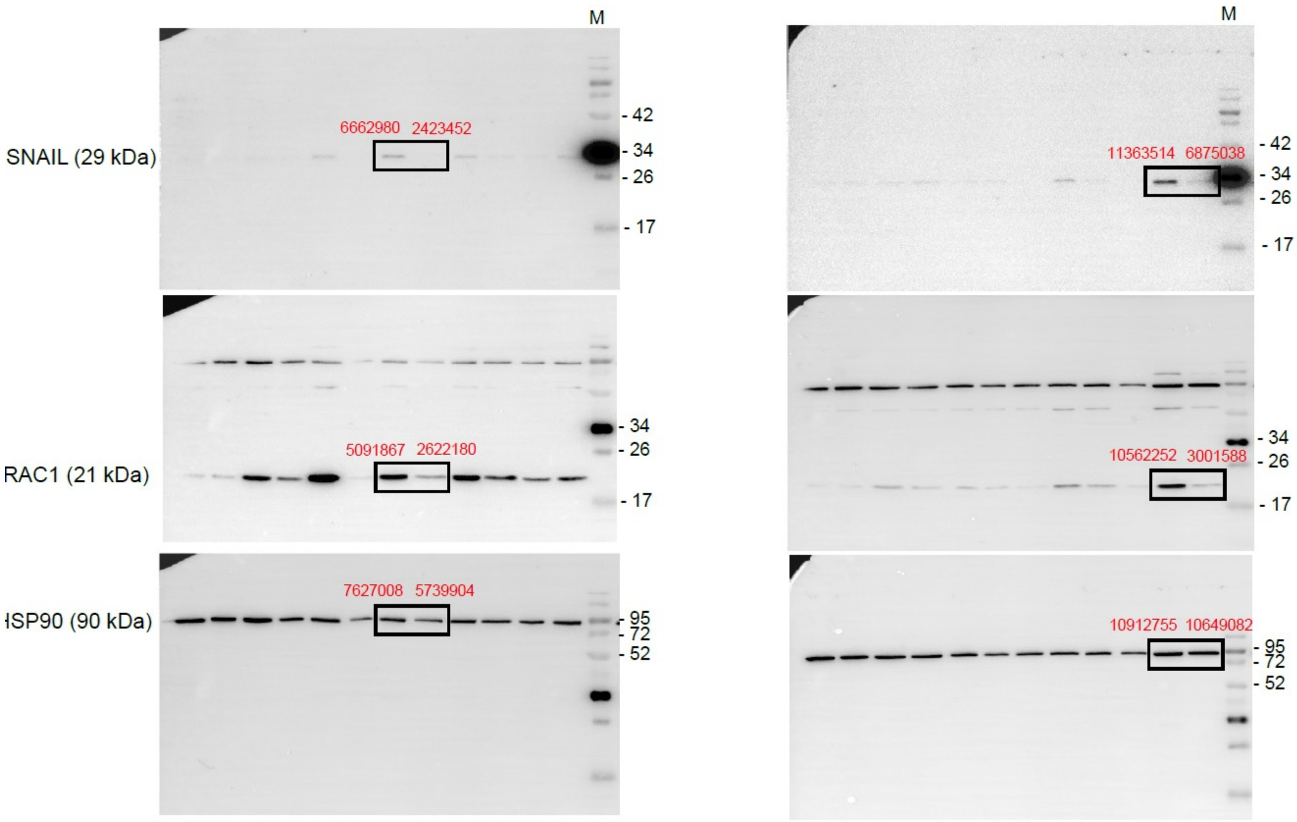

Figure S8. Uncropped blots of Figure 4B.

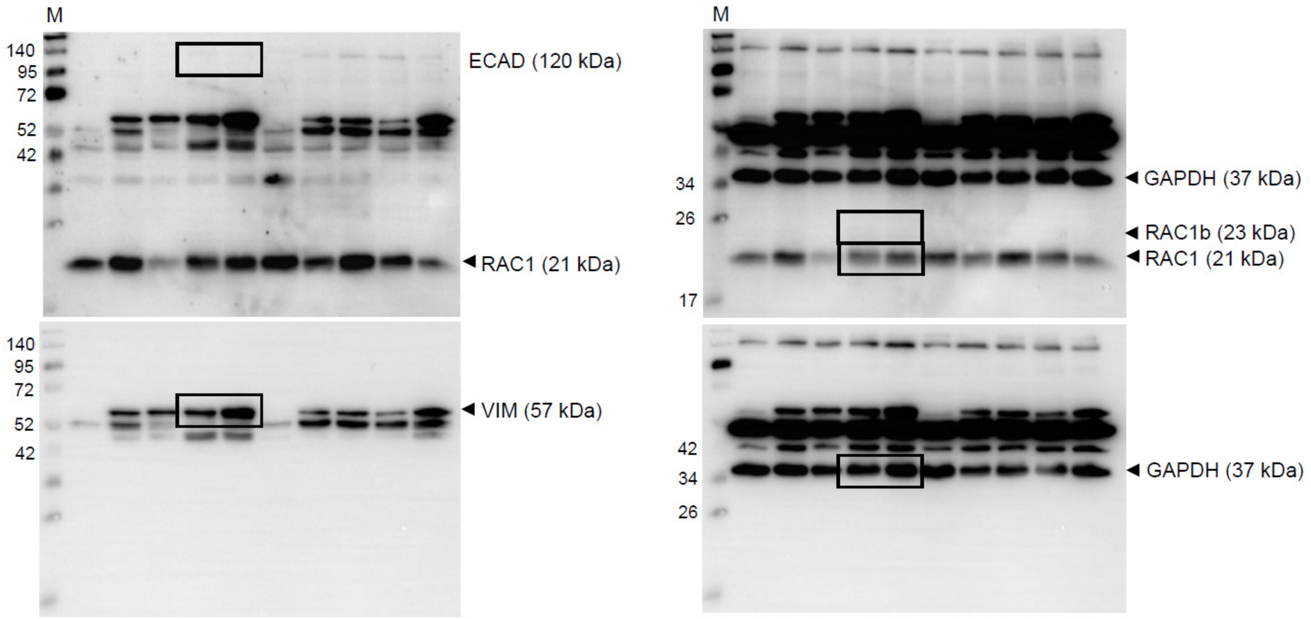

Figure S9. Uncropped blots of Figure S1A.

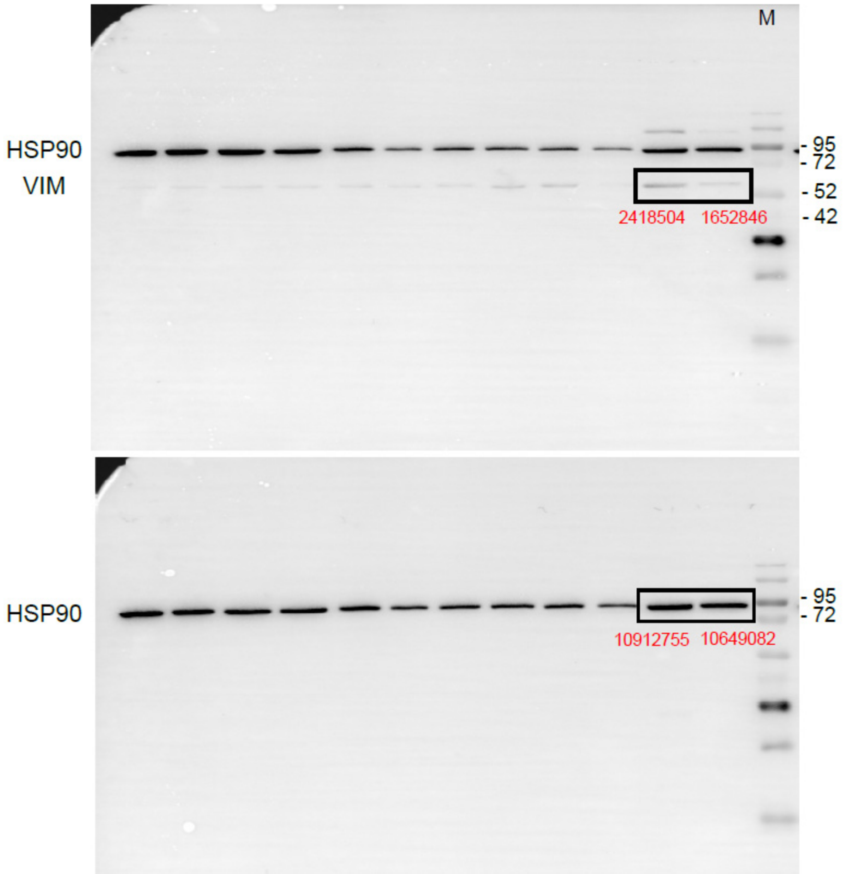

Figure S10. Uncropped blots of Figure S4.

Table S1. Primers used for qPCR.

Primer name	Sequence (5′→3′)	GenBank accession
Insulin-forward	gcagcctttgtgaaccaacac	NM_000207
Insulin-reverse	ccccgcacactaggtagaga	NM_000207
GAPDH-forward	ttgccatcaatgaccccttca	NM_001357943
GAPDH-reverse	cgcccacttgattttgga	NM_001357943
INSM1-forward	gtccacgcccgtttcctac	NM_002196
INSM1-reverse	ccaggttgaagctgcgttc	NM_002196
GLUT2-forward	ctgcattcagcaattggacctgc	NM_000340
GLUT2-reverse	tttacacagtctctgtagctcctag	NM_000340
NGN3-forward	ggcgctgactcaaacgctgc	NM_020999
NGN3-reverse	aagccagactgcctgggctc	NM_020999
NKX2.2-forward	aggaggcctcggtccttatgg	NM_002509
NKX2.2-reverse	agcgaagctgcgcaaacattctg	NM_002509
PAX6-forward	gaatcagagaagacaggccag	NM_000280
PAX6-reverse	gggtgtaggtatcataactccg	NM_000280
PAX4-forward	gaggacacggtgagggtctgg	AF043978
PAX4-reverse	aggggacagtgggaggaaggg	AF043978
MafA-forward	cgagctgaaccggcagctc	NM_201589
MafA-reverse	gccagcttctcgtatttctccttg	NM_201589
NeuroD1-forward	accatgaccaaatcgtacagcgag	NM_002500
NeuroD1-reverse	tggaagacatgggagctgtc	NM_002500
NKX6.1-forward	ccggacagcagatcttcgcc	NM_006168
NKX6.1-reverse	gacttgtgcttcttcaacagctgc	NM_006168
TBP-forward	gctggcccatagtgatcttt	NM_003194
TBP-reverse	cttcacacgccaagaaacag	NM_003194
Cytokeratin-19-forward	atggccgagcagaaccggaa	NM_002276
Cytokeratin-19-reverse	ccatgagccgctggtactcc	NM_002276
Claudin-7-forward	agctgcaaaatgtacgactcg	NM_001185022
Claudin-7-reverse	ggagaccaccattagggctc	NM_001185022
E-cadherin-forward	tcttccccgccctgccaatc	Z13009
E-cadherin-reverse	gcctctctcgagtcccctag	Z13009
EpCAM-forward	aatcgtcaatgccagtgtactt	NM_002354
EpCAM-reverse	tctcatcgcagtcaggatcataa	NM_002354

Table S2. Relative mRNA concentrations of epithelial genes in PDAC-derived cell lines. Data represent the mean ± SD of three samples taken at different time points during continuous cultures and analyzed by qPCR for expression levels of the indicated epithelial genes. Values of PANC-1 and COLO 357 cells are expressed relative to those of MIA PaCa-2 cells set arbitrarily at 1.0. Differences between MIA PaCa-2 and PANC-1, MIA PaCa-2 and COLO 357, and PANC-1 and COLO 357 are statistically significant for all three genes ($p < 0.05$, unpaired two-tailed Student's *t*-test).

	CLDN7	EpCAM	CDH1
MIA PaCa-2	1.0	1.0	1.0
PANC-1	13706 ± 981	77 ±11	647 ±86
COLO 357	20661 ± 1622	2370 ±196	32731 ±1976
